# Evaluating the Effects of Heat-Clearing Traditional Chinese Medicine in Stable Bronchiectasis by a Series of N-of-1 Trials

**DOI:** 10.1155/2022/6690638

**Published:** 2022-01-17

**Authors:** Suna Zi, Haiyin Huang, Peilan Yang, Minhua Xu, Yingen Wu, Zhenwei Wang, Fei Ge, Xinlin Chen

**Affiliations:** ^1^Yueyang Hospital of Integrated Traditional Chinese and Western Medicine, Shanghai University of Traditional Chinese Medicine, Shanghai 200437, China; ^2^Shanghai Guanghua Integrated Traditional Chinese and Western Medicine Hospital, Shanghai 200052, China; ^3^Longhua Hospital, Shanghai University of Traditional Chinese Medicine, Shanghai 200032, China; ^4^Basic Medical College of Guangzhou University of Traditional Chinese Medicine, Guangzhou 510006, China

## Abstract

**Purpose:**

The purpose of this study is to study the effects of heat-clearing Traditional Chinese Medicine (TCM) in the stable stage of bronchiectasis via N-of-1 trials.

**Methods:**

The N-of-1 trials in this study were randomized and double-blinded with crossover comparisons consisting of three pairs. Each pair was of two 4-week periods. Each patient took the individualized decoction in the experimental period and the individualized decoction was removed of heat-clearing drugs, mainly including heat-clearing and detoxifying drugs, in the control period for three weeks. After three weeks, the patients stopped taking the decoction for one week. The primary outcome was from patients' self-reporting symptoms scores on a 1–7-point Likert scale. Mixed-effects models were used to conduct statistical analysis on these N-of-1 trials.

**Results:**

Of the 21 patients enrolled, 15 completed three pairs of N-of-1 trials (71.43%). (1) Seen from the individual level, no statistical difference between the experimental decoction and the control (*P* *>* 0.05) was observed. However, 5 patients found better decoctions according to the clinical criteria. (2) As revealed by the group data of all the N-of-1 trials, the control was better than the individualized decoction in terms of symptom scores on the Likert scale (1.94 ± 0.69 versus 2.08 ± 0.68, *P* = 0.04, mean difference, and 95% CI: 0.19 (0.01, 0.37)) and on CAT scores (13.66 ± 6.57 versus 13.95 ± 6.97, *P* = 0.04, mean difference, and 95% CI: 0.86 (0.042, 1.67)), but such differences were not clinically significant. The other outcomes, such as Likert scale score of respiratory symptoms and 24-hour sputum volume, showed no statistical difference.

**Conclusion:**

The experimental design of this study can make the TCM individualized treatment fully play its role and can detect the individualized tendencies according to the severity of phlegm and heat in some subjects. With the intermittent use or reduced use of heat-clearing drugs, most of the subjects, at the group level, enrolled in the series of N-of-1 trials may improve the symptoms and quality of life while saving the cost of TCM and reducing the potential side effects of heat-clearing TCM. This trial is registered with clinicaltrials.goc (NCT03147443).

## 1. Introduction

Bronchiectasis is a common chronic lung disease characterized by heterogeneous clinical features and outcomes [1]. In Europe and North America, the prevalence of bronchiectasis ranges from 67/100,000 to 566/100,000, while in China it is as high as 1200/100,000, which thus brings a serious and growing economic health burden [2–4]. There are, throughout the stable stage of bronchiectasis, still chronic coughing, purulent sputum, breathlessness, and other clinical symptoms, as well as extrapulmonary symptoms including fatigue, insomnia, constipation, or diarrhea. Traditional Chinese Medicine (TCM) has been proven to be capable of easing the clinical symptoms, reducing the frequency of acute exacerbation every year, and improving the quality of life of patients in the stable stage of bronchiectasis [5]. In TCM, the main syndromes in the stable bronchiectasis are deficiency of both Qi and Yin, deficiency of lung-spleen Qi, and phlegm-heat syndrome [5–7]. Therefore, the basic therapeutic principles include strengthening the body's resistance by replenishing Qi, nourishing Yin, invigorating the spleen, removing phlegm, and clearing heat. Wu and Tang [8] considered that “phlegm-heat” syndrome is the most common clinical syndrome of bronchiectasis, thus indicating that “phlegm-heat” is the principal contradiction in the treatment of bronchiectasis. It is generally known that patients with bronchiectasis mainly cough up yellow sputum and are prone to hemoptysis. While heat-clearing and phlegm-transforming drugs, or blood-cooling and hemostatic drugs, are recommended, tonics or warming drugs should be used with caution to avoid furtherance of both heat and phlegm. Tian Zhengjian pointed out that [9] heat-clearing drugs should be administered no matter in the acute exacerbation or stable stage of bronchiectasis. However, according to the theory of TCM, such heat-clearing drugs should not be used excessively or in a protracted period, for fear that bitter dryness hurts Yin and coldness spoils the stomach which will then injure the spleen and inhibit the movement of Qi [8]. While strengthening the body's resistance and removing phlegm are generally regarded as the two therapeutic principles in the stable bronchiectasis, there has been controversy over the role and course of heat-clearing Traditional Chinese Medicine (TCM) in this stage. Microorganisms including *Pseudomonas aeruginosa* and other Gram-negative and Gram-positive microorganisms (to a lesser extent) identified in the culture are related to disease progression, adverse clinical outcome of bronchiectasis, and inflammation mediated by neutrophils in the driving airway [10]. Most heat-clearing and detoxifying Traditional Chinese Medicines have been proven to have certain antibacterial effects by clinical or in vitro experiments [11]. For example, Zaiyuan Pang and colleagues found that *Scutellaria baicalensis*, Forsythiae Fructus, *Coptis chinensis*, and honeysuckle have different degrees of bacteriostasis in vitro against widely resistant *Pseudomonas aeruginosa* [12]. Thus, it is important to investigate the function of heat-clearing TCM in the stable stage of bronchiectasis.

There was no randomized controlled comparison between the efficacy and side effects of decoctions containing heat-clearing drugs and decoctions removed of heat-clearing drugs in the stable stage of bronchiectasis. N-of-1 randomized controlled trials (“N-of-1 trials”) were conducted with the subject himself/herself as the control. In a prospective, single-individual, planned, multiple-crossover trial, the patient undergoes paired treatment periods that are organized such that one period of each pair adopts the experimental therapy and the other period uses either an alternative treatment or placebo. The 2-cycle sequence in each pair is random. The individualized treatment concept represented by N-of-1 trials naturally conforms to the principle of syndrome differentiation in TCM (which must be applied individually) and provides a feasible way for the integration of TCM and Western medicine. Our previous studies have demonstrated a trend of beneficial effects of individualized herbal decoction compared to standard decoction through a series of N-of-1 trials [13]. However, the possibility of “carryover effects” reduces the reliability and sensitivity of the N-of-1 trials for TCM. In this study, we planned to use a mixed-effects model that accurately estimates the treatment effects and controls the “carryover effects” [14,15]. Given this, we intended to identify the role of heat-clearing drugs in the syndrome differentiation and treatment of bronchiectasis in its stable stage through a series of N-of-1 trials.

### 1.1. Hypotheses and Objective

It is predicted, on the group level, that the individualized decoction is more effective than the individualized decoction removed of heat-clearing drugs. However, at the individual level, the effect may vary with the severity of phlegm and the heat of the individual. The effects of individualized treatment based on TCM syndrome differentiation will be reflected. The results may have the guiding value for TCM clinical practice.

## 2. Methods

### 2.1. Study Design

The study design of these N-of-1 trials have been developed according to the literature of Guyatt et al. [16,17] and described in our previous study [13,18]. Briefly, the patients who met the inclusion criteria and received open preliminary trial treatment could be enrolled into the N-of-1 trials. During the run-in period, we got the onset time after drug administration and the efficacy maintenance time after drug withdrawal for the purpose of determining the length of the observation period and estimating the washout period. The observation period of the N-of-1 trials was set to be four weeks based on a comprehensive analysis of the results in the run-in periods and the results previously obtained [13,18].

**Figure 1:**
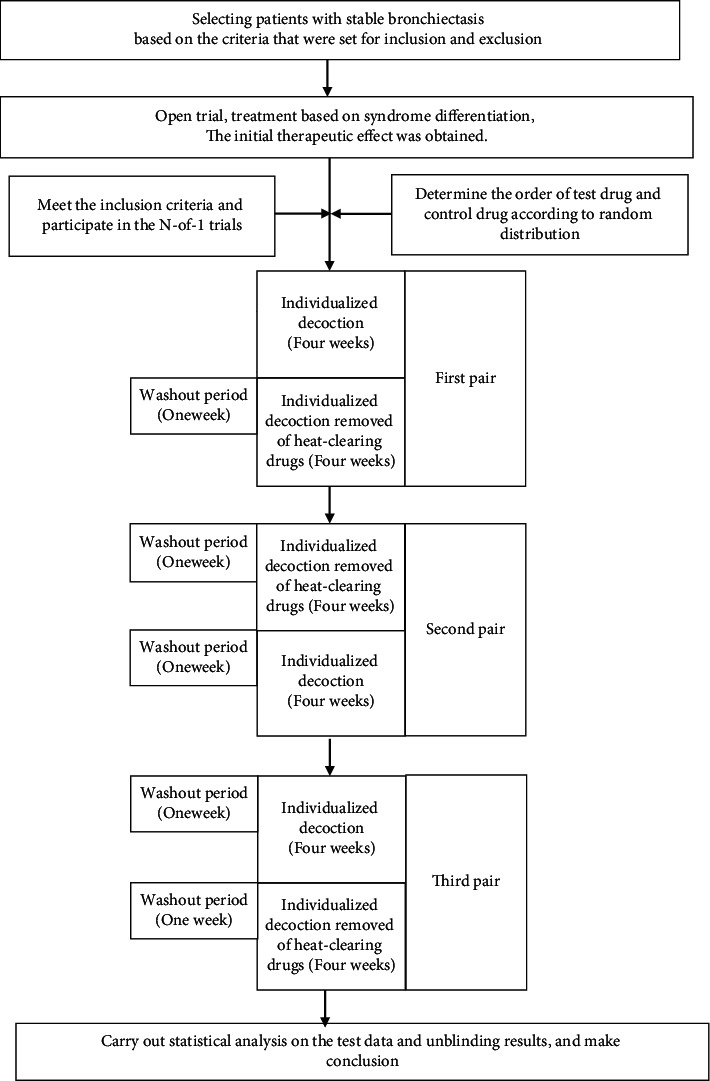
The flow chart of the N-of-1 trial of an individual patient in this study.

We conducted three pairs of N-of-1 trials in one individual, and each pair consisted of two observation periods, that is, the experimental period and the control period, which were assigned randomly. Each observation period lasted for four weeks, and the medications were taken for three weeks. The medications were then stopped for one week at the last week of each observation period. The outcomes in this week were measured in each period. The washout period is the time before the measured week (fourth week) (Figure 1).

When the patients have acute exacerbations during the N-of-1 trials, they can receive antibiotics and other treatments routinely, and the data of this pair would not be statistically analyzed [19]. The study resumed when the disease returned to a stable stage. If a patient felt worse at any time during the trial, the current treatment period was terminated and, without breaking the blinding, the next treatment period began [17].

### 2.2. Patients and Diagnosis

#### 2.2.1. Inclusion Criteria

1. In line with the diagnostic criteria of bronchiectasis in accordance with the consensus of Chinese experts [20] and the guidelines for noncystic fibrosis bronchiectasis published by the British Thoracic Society in 2010 [21]
2. Age of 18–70 years
3. In the stable stage of bronchiectasis and no acute exacerbation within 3 weeks
4. Frequency of acute exacerbation of bronchiectasis ≤3 times every year
5. Signing informed consent

The diagnostic criteria for TCM syndrome differentiation were established in accordance with the “Criteria of Diagnosis and Therapeutic Effect of TCM Diseases” [22] and in combination with the research literature on the TCM syndrome differentiation rule of bronchiectasis [6]. It mainly includes lung and spleen deficiency syndrome, Qi and Yin deficiency syndrome, and phlegm-heat obstructing lung syndrome. Two main symptoms or more than two simultaneous symptoms showing corresponding tongue and pulse signs can constitute the TCM syndrome of each patient.

Clinically, the TCM syndrome differentiation of stable bronchiectasis is mostly the mixture of deficiency and excess of a certain syndrome type. There is a certain degree of phlegm and heat in each syndrome type of TCM. For example, for the syndrome of deficiency of both Qi and Yin combined with phlegm and heat syndrome or lung and spleen deficiency with phlegm and heat syndrome [5,8], the severity of intermingled phlegm and heat should be estimated based on clinical experience. In order to make sure that the treatment based on syndrome differentiation of TCM was accurate and effective, two chief physicians were invited to evaluate and conclude the TCM syndrome of each subject. In case of need, a third party would be invited (a distinguished veteran doctor of TCM).

The trial was carried out at the clinic of Yueyang Hospital of Integrated Traditional Chinese and Western Medicine, Shanghai University of Traditional Chinese Medicine.

### 2.3. Randomization and Blinding

A randomized block design was adopted in this study, and the order of medication for three rounds of subjects in each single case trial was determined according to the random number generated by SPSS software, with a block number of 2 [13], such as AB-BA-AB or BA-BA-AB. First, the doctor simultaneously prescribed individualized decoction and control decoction. Then, the observer gave the random medication order together with the prescriptions to a specifically designated pharmacist in the TCM pharmacy. The pharmacist tossed a coin to determine which of A or B represented the individualized decoction or the control decoction and then recorded the blind code and kept it private. According to the random order of the subjects' medication, the herbal medicines were prepared according to the prescription and sent to the decoction room of the hospital for decoction. Finally, the drug dispensers distributed the drugs to the subjects and were responsible for medication records to ensure that drug trials were blind between doctors and patients.

### 2.4. Interventions

Chest physiotherapy was the primary treatment for stable bronchiectasis, which could promote the excretion of respiratory secretions. If the patient has complications with coronary heart disease, diabetes, or hypertension, the medicine for the concomitant diseases could be taken simultaneously. However, the medication should be relatively fixed, and the dosage and the administration of the medicine should be consistent with the experimental period and the control period of each cycle, as well as a detailed medication administration record.

#### 2.4.1. Syndrome Differentiation Decoction (Individualized Decoction) Adopted in the Tested Drug Observation Period

In accordance with the therapeutic principles of strengthening body resistance, replenishing Qi, nourishing Yin, invigorating spleen, removing phlegm, and clearing heat, we prescribed the individualized decoction based on Bronchiectasis Stabilization Decoction [13] (Radix Lithospermi 15 g, Rhizoma Fagopyri Cymosi 30 g, Radix Ophiopogonis 15 g, Poria 15 g, Astragalus Astragali 20 g, Rhizoma Bletillae 10 g, *Platycodon grandiflorum* 10 g, and Semen Coicis 30 g). For subjects having the syndrome of lung and spleen Qi deficiency, Radix Codonopsis Pilosulae, Pericarpium Citri Reticulatae, and *Atractylodes macrocephala* Koidz. were added. For subjects having the syndrome of Qi and Yin deficiency, Radix Adenophorae, Radix Glehniae, and Radix Rehmanniae were added. For syndromes intermingled with phlegm and heat, we added heat-clearing and detoxifying drugs (e.g., *Houttuynia cordata*, *Viola mandshurica*, and Dandelion), heat-clearing and damp-drying drugs (e.g., *Scutellaria baicalensis* and *Coptis chinensis*), and heat-clearing and fire-purging drugs (e.g., Gardenia), depending on the clinician's estimation of the severity of intermingled phlegm and heat. Apart from that, decoctions can also be adjusted according to other symptoms of the subjects, such as constipation, diarrhea, fatigue, and insomnia. Besides, we also adjusted the individualized decoction in accordance with the change in the patient's condition throughout the study.

#### 2.4.2. The Individualized Decoction Removed of Heat-Clearing Drugs (Control Decoction) Adopted in the Control Drug Observation Period

The heat-clearing TCM were removed from the individualized prescription as the control. The heat-clearing TCM include heat-clearing and detoxifying drugs (e.g., *Houttuynia cordata*, *Viola mandshurica*, and Dandelion), heat-clearing and damp-drying drugs (e.g., *Scutellaria baicalensis*), certain heat-clearing and blood-cooling drugs (e.g., Arnebia), and heat-clearing and fire-purging drugs (e.g., Gardenia). However, Radix Rehmanniae Recen and Radix Scrophulariae were not removed as heat-clearing and blood-cooling drugs due to their effect of nourishing Yin and generating Jin. Besides, reed root as medicine for clearing heat and purging fire was not removed, because it is good at nourishing Yin and quenching thirst. It is also an essential part of Qianjin reed stem soup, which is a prescription commonly used for the treatment of bronchiectasis.

In an observation period, both of the decoctions were taken for three weeks, and each was stopped for one week.

#### 2.4.3. Herbal Preparation and Quality Assurance

The decoction of TCM was formulated according to the literature [23]. The traditional Chinese medicinal herbs which satisfied the national norms [18] were provided by the Shanghai Tongjitang Pharmaceutical Co., Ltd. The pieces of herbs were soaked in water for 30 min in nonwoven bags and decocted 1 time for 30 min at 110°C (pressure 0.1 MPa) in a TCM decocting machine produced by Beijing Donghuayuan Medical Equipment Co., Ltd. (model: YJ 20-G). The Chinese herbal decoctions were taken by one decoction a day and divided into 2 doses.

### 2.5. Outcome Measures

The doctors in charge of the treatment visited the patient and collected data prior to and after each treatment period. Subjects identified the symptoms that bothered them and filled out patient diaries or questionnaires every day. The outcome measures are listed below.

#### 2.5.1. Primary Outcome: Patient Self-Rated Symptom Score (7-Point Likert Scale)

The patients were assessed for the severity of the symptoms (cough, expectoration, shortness of breath, chest pain, loss of appetite, fatigue, insomnia, and so on) using the 7-point Likert scale [13,16]. It is necessary to optimize the number of questions to make sure that the most important aspects of the patient's problem are examined (usually four to eight items). Each patient was graded daily for the severity of the problems on a 7-point Likert scale supplemented by Visual Analogue Scales (VAS). A higher score indicates more serious symptoms.

A 0.5 improvement in each question indicates a significant improvement in the patient's well-being. If there are seven questions, then in this case a total change of 3.5 or more points is considered to have clinical significance [16,17]. Therefore, the average difference of 0.5 points was defined as the “Minimal Clinically Important Difference (MICD)” for the 7-point scale.

#### 2.5.2. Other Outcomes

*(1) 24 h Sputum Volume*. The average value of the 24-hour sputum volume for three consecutive days, that is, the three days before the start of the trial regimen or three days before the end of each observation period during the trial, was taken. In order to ensure the accuracy of the measurement of sputum volume, the subjects were instructed to spit the sputum into a special sputum cup with a scale of 100 mL from 8 am on the first day to 8 am the next day and finally recorded the sputum volume in the diary.

*(2) COPD Assessment Test (Chronic Obstructive Pulmonary Disease Assessment Test, CAT)*. The CAT questionnaire consists of 8 questions. The score range for each item is from 0 to 5, so the range of the total score is from 0 to 40. The score of 0 denotes the best quality of life, and the score of 40 represents the worst quality of life. CAT was originally designed for evaluating the quality of life of chronic obstructive pulmonary disease patients. Recently, Lee [24] confirmed that CAT was valid and reliable in evaluating the quality of life of bronchiectasis patients. The MCID (the Minimal Clinically Important Difference) for the CAT has not been formally established, but it was estimated at approximately 2 points [13,25].

*(3) TCM Syndrome Scores.* In reference to “Guiding Principles for Clinical Research of New Drugs of Traditional Chinese Medicine” [26], the clinical TCM syndromes including cough, expectoration (color, quality, and quantity), hemoptysis, wheezing, fatigue, anorexia, dryness of mouth and throat, spontaneous sweating, night sweating, tongue coating (tongue body), and pulse condition were graded before trials and at each time point after treatment.

*(4) Safety Outcome*. Blood and urine routine, liver and kidney function, electrocardiogram, and so forth were measured prior to and after the trial, trial-related adverse events were observed, and the trial was unblinded or discontinued as necessary.

### 2.6. Data Analysis

#### 2.6.1. Sample Size Calculation

The main results of the study were patients' self-reported symptom scores on a 1–7-point Likert scale. The amount of samples required was estimated on the premise of having at least 80% power (*β* = 0.20) to detect a mean difference of 0.5 points (the “Minimal Clinically Important Difference (MICD)) in Patient Self-Rated Symptom Score, with a significance test at the *α* = 0.05 level. The standard deviation (SD) of the data from this study was 0.53. Using a two-sided test, it was assumed that there was no period effect or treatment × time interaction under the given model parameters, so the ratio of the two groups was 1 : 1, with three cross-overs [27,28]. The sample size was calculated using the PASS 11.0 software (NCSS LLC, Kaysville, UT, USA). The result indicated that it took 12 patients to meet the same significance and power requirements. The final sample size was set as 16 due to the high drop-out rates of N-of-1 trial (30%).

#### 2.6.2. Statistical Analysis

Aiming to reduce autocorrelation (that is, for data that are not independent) [29], the mean values of the data were collected from the last week of each observation period. SAS 9.4 (SAS Institute, Cary, NC) was adopted to conduct statistical analyses. For the data of normal distribution, paired *t*-test was used for single case and mixed-effects model for N-of-1 trials as a group. When there was a carryover effect, the mixed-effects model could be adopted to accurately estimate the treatment effects and control carryover effects [14,15]. In the construction of mixed-effects model, the intervention effect, stage effect, and residual effect were regarded as fixed effects, and the subject was regarded as random effects. If the stage effect and residual effect in the fixed effects were not significant, then one effect was deleted in turn until the result had statistical significance. If there was no statistical significance, only the intervention effect and the random effect of the subject were included for analysis. If the *P* value was smaller than 0.05, it was statistically significant for each test.

#### 2.6.3. Clinical Efficacy Criteria

In order to make up for the limited power of statistical tests of individual N-of-1 trial, we also adopted the Clinical Efficacy Criteria proposed by Guyatt et al. for the definite answer of N-of-1 trial [17] (see Table 1).

### 2.7. Ethics

The trial protocol was approved by the Ethics Committee of Yueyang Hospital of Integrated Traditional Chinese and Western Medicine, Shanghai University of Traditional Chinese Medicine (ethical review approval number: 2016-103). Volunteers from Shanghai city were recruited via advertisements and medical lectures. The informed consent of all subjects was obtained.

## 3. Results

### 3.1. General Information of the Study

**Figure 2:**
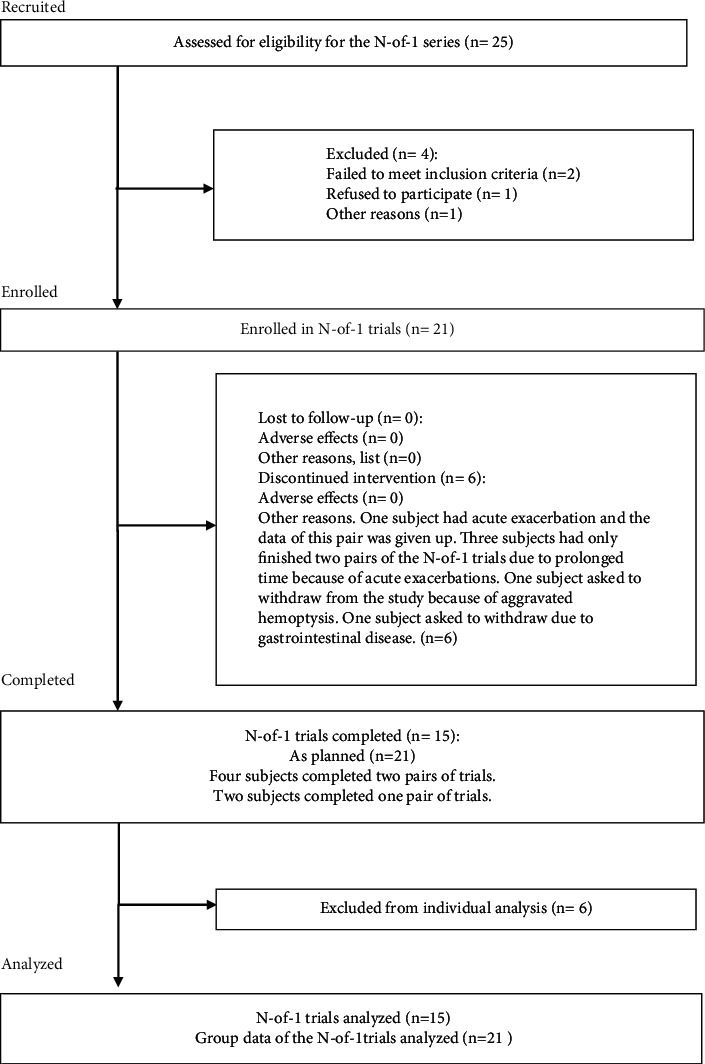
The flow chart of the whole process including the number of cases recruited, enrolled, and completed in this study.

From May 2016 to Oct. 2017, patients were recruited for this study in the Clinic of Yueyang Hospital of Integrated Traditional Chinese and Western Medicine, Shanghai University of Traditional Chinese Medicine. A total of 25 patients with bronchiectasis satisfied the inclusion criteria, and 21 subjects were formally enrolled in this study and signed the informed consent forms. 15 of these patients (71.43%) completed the N-of-1 trials (71.43%), including three pairs of randomized, double-blind, and controlled trials. 4 subjects completed two pairs (19.05%), and 2 subjects completed only one pair (9.52%). The flow chart of the study and the reasons for withdrawal and exclusion are shown in Figure 2. The baseline data of the 21 subjects formally enrolled are listed in Table 2.

### 3.2. The Results of the Individual Data of the N-of-1 Trials

#### 3.2.1. Results Based on Statistical Criteria

Nearly all of the outcomes, including the self-reported symptom scores on Likert scale, 24-hour sputum volume, CAT scores, and TCM syndrome scores, showed no statistical difference (*P* > 0.05) between individualized decoction based on syndrome differentiation and individualized decoction removed of heat-clearing drugs (Table 3).

#### 3.2.2. Results Based on Clinical Efficacy Criteria

A total of 5 subjects identified the preferable decoctions according to Clinical Efficacy Criteria (Section 2.6.3), among which 4 subjects preferred individualized decoction and 1 subject preferred individualized decoction removed of heat-clearing drugs.

**Case 4 .**

(female). Syndrome differentiation indicated deficiency of both Qi and Yin, with obvious phlegm-heat. She felt the efficacy varied with each period of medication. The individualized decoction was superior to the individualized decoction removed of heat-clearing drugs after unblinding. The clinician judged that individualized decoction was better.

**Case 7 .**

(female). Syndrome differentiation indicated deficiency of both Qi and Yin, combined with mild phlegm-heat. She felt that the efficacy varied with each period of medication. The individualized decoction removed of heat-clearing drugs was superior to the individualized decoction after unblinding. The clinician judged that the individualized decoction removed of heat-clearing drugs was better.

**Case 10 .**

(female). Suffering from cough and a large amount of yellow sputum with a history of repeated hemoptysis, this case had the syndrome differentiation of Qi and Yin deficiency with heavy phlegm-heat. At the end of the first period of the second pair, massive hemoptysis suddenly occurred and the subject withdrew from the trial. The unblinding results showed that the patient had been given individualized decoction removed of heat-clearing drugs for nearly two consecutive periods in a random order. The etiology of hemoptysis may be due to the heavy phlegm-heat in the subjects and the long-term discontinuation of heat-clearing drugs. Since then, she has been given heat-clearing drugs to avoid using warm and dry TCM in her prescriptions.

**Case 12 .**

(female). This case had the syndrome differentiation of deficiency of Qi and Yin combined with obvious phlegm-heat, together with a history of hemoptysis. The subject perceived differences in the efficacy over the different periods of the N-of-1 trials. It was confirmed after unblinding the effect of the individualized decoction was superior to that of the individualized decoction removed of heat-clearing drugs. This case met the Clinical Efficacy Criteria.

**Case 13 .**

(female). The subject often had bloody sputum. She had the syndrome differentiation of deficiency of Qi and Yin combined with obvious phlegm-heat. After unblinding, the subject was found to be more inclined to individualized decoction in terms of hemostatic effect.

### 3.3. The Results of the Group Data

#### 3.3.1. Comparison Results of Group Data between the Two Decoctions in Each Observation Outcome

We adopted the mixed-effects model to carry out the analysis. The intervention effect was statistically significant for both the self-reported symptom scores on the Likert scale (mean difference 0.19; 95% CI: 0.01, 0.37; *P* = 0.04) and the CAT scores (mean difference 0.86; 95% CI: 0.042, 1.67; *P* = 0.04), suggesting that the individualized decoction removed of heat-clearing drugs was superior to the individualized decoction on the two outcomes but not clinically significant (MCID < 0.5 on Likert scale score of symptoms and MCID< 2 on CAT scores, respectively). The other outcomes (Likert scale score of respiratory symptoms, 24-hour sputum volume, and TCM syndrome scores (including Qi and Yin deficiency syndrome and lung and spleen Qi deficiency syndrome) showed no statistical difference. Details are shown in Table 4.

### 3.4. Safety Outcome

Gastrointestinal reaction occurred in the third pair of Case 14, and the patient recovered after one week of drug discontinuation, which was considered to be related to heat-clearing drugs after unblinding. Serious adverse events did not occur throughout the study. The results of blood and urine tests including liver and kidney functions were normal prior to and after the N-of-1 trials were normal.

## 4. Discussion

### 4.1. Summary of the Results in This Study

#### 4.1.1. Summary of the Results of the Individual Data

Although there was no statistically significant difference on the individual level between the individualized decoction based on syndrome differentiation and the individualized decoction removed of heat-clearing drugs (*P* > 0.05), this did not mean that there was no difference between the two decoctions on individual level. Because a single N-of-1 trial had only a limited number of pairs (≤3), its statistical ability was low. Guyatt et al. thought that “using N-of-1 trials to enhance patient care is not dependent on the statistical analysis of the results.” Therefore, they proposed the Clinical Efficacy Criteria for N-of-1 trials: “The N-of-l trial was interrupted before the completion of three treatment pairs because the clinician believed that the efficacy of the drug had been established or disproved (perceived greater therapeutic efficacy or severe side effects, both of which were confirmed after noncompliance)” [16,17]. From the perspective of individual analysis for each patient, there appeared some tendencies. A total of five subjects found out which of the two decoctions was better in accordance with clinical criteria. Among them, four were more inclined to the individualized decoction based on syndrome differentiation (A), and one was more inclined to the individualized decoction removed of heat-clearing drugs (B). While the four who were inclined to A were characterized by severe phlegm and heat as well as frequent hemoptysis, the one who was inclined to B was mild in terms of phlegm and heat.

#### 4.1.2. Summary of the Results of N-of-1 Trials

Based on the analysis of group data, the results of this study were inconsistent with the original assumption (the hypothesis of the study). It is believed that this situation may not be uncommon with the further development of N-of-l trials of TCM. The scores of self-reported symptoms and CAT of patients taking individualized decoction removed of heat-clearing drugs were better than those taking the individualized decoction (*P* < 0.05). However, the difference in Likert scale score for the overall symptoms between the two decoctions was only 0.19, which was not clinically significant. The other outcomes (Likert scale score of respiratory symptoms, 24-hour sputum volume, and TCM syndrome scores) showed no statistical difference.

We also observed whether the sequence of the first pair of medication would have an impact on the results. The results indicated that there was no statistically significant difference in the Likert scale score of self-evaluated overall symptoms between the two groups (*P* > 0.05). The disadvantage is that the number of cases is limited.

### 4.2. Comparing Findings with Other Studies

In recent years, there has been an increasing trend in the publication of the literature on N-of-1 trials of Traditional Chinese Medicine (TCM) at home and abroad, which indicates that it is feasible for the trials to be designed for clinical research of TCM and that it also demonstrates the features of treatment based on syndrome differentiation [30–35]. The number of cases in each study varies from 1 to 24 cases, with the observation period from 2 to 4 weeks and the administration time of Traditional Chinese Medicine from 3 days to 3 weeks. Many trials were designed to set a one-week washout period between the administration of the trial drugs and that of the control drugs. The outcome measures include TCM syndrome score, quality of life scale, or laboratory outcomes such as hemoglobin and platelet count. More studies have used blinding rather than blank control, indicating that the quality of N-of-1 trials of TCM is improving. Most of the studies showed that the treatment of TCM was effective (*P* < 0.05). However, some studies only carried out the group-based statistical analysis instead of individual analysis and statistics. Some of the studies used improper statistical methods, such as the use of the *t*-test in individual or group statistics.

In this study, the combination of individual and group analyses has been applied to enhance the sensitivity of N-of-1 trials of TCM. On the basis of the previous study [13], we reduced the medication duration of Traditional Chinese Medicine in each period from 4 weeks to 3 weeks, leaving the remaining one week as the washout time without medication, so that the whole washout period was actually prolonged to 4 weeks. In addition, we used the mixed-effects model for group-based statistical analysis, taking into account the possible stage effects and residual effects. The result showed that stage effect and residual effect had no statistical significance (*P* > 0.05).

### 4.3. Strengths and Limitations

#### 4.3.1. Fully Utilizing the Individualized Treatment Based on Syndrome Differentiation

Since the design of the N-of-1 trial can be highly individualized, patients with different TCM syndromes or mixed syndromes could be included in the trial. While subjects received individualized TCM treatment based on syndrome differentiation, the prescription could also be adjusted according to the changes of symptoms or syndromes throughout the trial, which is identical to the real clinical practice. This is one of the most prominent superiorities of N-of-1 trials in the study of TCM.

#### 4.3.2. Improving the Efficiency of Blind Method in the Clinical Trials of TCM

In this study, each subject was required to receive the intervention of two kinds of TCM decoctions at random, which made a high demand on blinding. However, due to the unique perception, taste, and order of TCM, finding a control drug that is completely consistent with the test drug can be extremely difficult [36]. In this study, the two TCM decoctions could be similar in appearance and size, but there may still be slight differences in taste and smell. To compensate for this difference, we informed subjects that both the test and control decoctions were likely to be effective regardless of taste and odor. This strategy proved effective in practice. Even if there were differences, most subjects did not know what type of decoction they were assigned to and most subjects did not show a preference for certain decoctions. The results of this study were not consistent with the initial expectations: no statistically significant difference between the experimental decoction and the control was observed at the individual level, and the control decoctions were even better in some outcomes (patient self-reported symptom scores on Likert scale and CAT scores) on the group level. Therefore, the data of this study has a relatively high degree of objectivity. We think that this strategy may be beneficial to improve the blinding of N-of-1 trials of TCM in the near future.

#### 4.3.3. Fewer Interference Factors of Western Medicine

The treatment of Western medicine is essential in a considerable number of clinical studies of TCM. Still, it is difficult to avoid a certain degree of interference even if the control group is treated with the same Western medicine treatment. In this study, most of the subjects were patients with mild-to-moderate severity of bronchiectasis, so the basic treatment was expectoration or postural drainage. There is generally no use of Western medicine with the exception of rare cases. Therefore, fewer interference factors of Western medicine improve the reliability of this study.

#### 4.3.4. Sensitivity Needs to be Further Improved

Because of the longer trial pair of the N-of-1 trials of TCM (about 8 weeks per pair), each case took about half a year, so usually only three pairs can be completed. For individual statistics, the statistical power was limited, and it was difficult for some patients to draw definite conclusions. The classic N-of-1 trials require the drugs studied to take effect quickly and have a short half-life [16,17], but the determination of the process of Chinese medicine metabolism can be very difficult. We had to run a preliminary trial, in conjunction with the investigators' clinical experiences, to identify the observation period and the washout period [18]. The relatively longer observation period is also a common weakness in N-of-1 trials of TCM.

### 4.4. Implications for Clinical Practice and Future Research

#### 4.4.1. Repositioning the Effect of Heat-Clearing Drugs in the Stable Stage of Bronchiectasis

*(1) Intermittent Application of Heat-Clearing Drugs May Be Better*. The most common TCM syndrome type of bronchiectasis is phlegm and heat obstructing the lung [3,4], indicating that “phlegm and heat” is an essential factor in the pathogenesis of bronchiectasis. Although the role of heat-clearing drugs is beyond all doubt, there is a lack of in-depth evidence-based medicine research on the application rules of heat-clearing drugs under the conditions of intermingled deficiency and excess as well as individual differences in the stable stage of bronchiectasis. According to our clinical experience in the stable stage of bronchiectasis, we tend to strengthen the body resistance while keeping in mind to clear lungs and resolve phlegm with both clearing and tonifying methods. However, Hong Guangxiang [37] believes that the asthenia in origin caused by bronchiectasis is dominated by the weakness of Qi and Yang and that phlegm is essentially the wet phlegm, which is the evil of Yin that cannot be removed without warmness. “The treatment of lungs does not reject warm drugs.” The continuous application of heat-clearing drugs may cause gastrointestinal reactions and also have potential hepatorenal toxicity.

The results of this study, to a certain extent, have weakened the role and positioning of heat-clearing drugs in the stable stage of bronchiectasis. In accordance with the theory of TCM, the prolonged application of heat-clearing drugs may lead to the impairment of stomach due to bitter cold, the hindrance of the movement of Qi, the injury of Yin due to bitter dryness, and so on.

However, since phlegm and heat still exist in the stable stage of bronchiectasis, it is not practical to abandon heat-learning Traditional Chinese Medicine in the stable stage of bronchiectasis. In this study, the alternating application of individualized decoction on the basis of syndrome differentiation and the individualized decoction removed of heat-clearing drugs has achieved favorable clinical effects in most patients, which suggests that, in the stable stage of bronchiectasis, it is not necessary to use heat-clearing drugs in the whole process of the treatment and that intermittent use of the same may be taken into account. This has a certain clinical reference value for reducing the potential side effects of heat-clearing drugs as well as the cost of TCM.

However, individualized treatment should always be applied to the clinical practice. Heat-clearing drugs should still be used in large dose or prolonged period for those bronchiectasis patients who have heavy phlegm-heat syndrome or are prone to hemoptysis.

*(2) Enlightenment on the Course of Treatment of TCM in the Stable Stage of Bronchiectasis*. At present, there is a lack of in-depth and systematic study on the treatment courses of chronic diseases such as bronchiectasis in TCM as well as on the safety of long-term medication. In TCM medication practice, three months can be used as one course of treatment, or two courses can be used consecutively [2]. In addition to the current doubts about the safety of long-term application of TCM (including heat-clearing drugs such as *Houttuynia cordata* Thunb) at home and abroad, quite a few of TCM medications in clinical practice adopt a dose in excess of that recommended in Chinese Pharmacopoeia, along with large prescriptions and long courses of treatment from time to time, which not only increases medical costs but also leaves safety loopholes. Attention must be paid to this issue. We set each observation period of N-of-1 trials to 4 weeks, including a medication period of 3 weeks and a suspension of 1 week. After that, the medication is rotated. Our original intention was to extend the washout period. However, in this study, it has been widely recognized by the subjects. They believed that a week of suspension did not reduce efficacy but instead gave them a short rest during the half-year trial. Compared with the traditional continuous use of Traditional Chinese Medicine [5], this medication method may reduce the side effects and the costs of TCM, which unintentionally offers enlightenment on the clinical medication practice for bronchiectasis or other chronic diseases.

#### 4.4.2. Exploring a More Efficient Statistical Method Suitable for N-of-1 Trials of TCM

In this study, although 5 subjects had found out which of the two decoctions was better in accordance with clinical criteria, no statistical difference can be drawn from any individual case, indicating that the sensitivity of this statistical methodology needs to be further improved. Thanks to its remarkable characteristics, hierarchical Bayesian statistical method is an important statistical method in N-of-1 trials [38]. The Bayesian method has the following advantages over the frequentist statistical methods: (1) it can simultaneously carry out the integrated analysis of individual and group data; (2) it is easy to introduce confounding variables, like the physique or gene type of different subjects, or different TCM syndromes (which helps to distinguish between the different TCM syndromes and differences in the effects); (3) if many patients have completed similar N-of-1 trials and the variance within an individual patient is greater than that between patients, the results of other patients can be used to enhance the accuracy of an individual result, meaning to increase the sensitivity of N-of-1 trials without increasing the pairs of N-of-1 trials. Currently, although this statistic method is rarely used in N-of-1 trials of TCM, it can be used for reference in our future study [39,40].

In summary, the effect of TCM individualized treatment in the experimental design in this study can be fully mobilized. The experimental design was able to detect the individualized tendency depending on the severity of phlegm and heat in some subjects. At the group level, for most of the subjects enrolled in the series of N-of-1 trials, intermittent use or reduced use of the heat-clearing drugs may improve the symptoms and quality of life, while saving the cost of TCM and reducing the potential side effects of heat-clearing TCM. More cases are needed to further substantiate the supposition.

## Figures and Tables

**Table 1:** Clinical criteria for definite N-of-1 trial.

1.	Clinicians have high confidence in the decisions made after the N-of-1 trials (1 or 2 on a 7-point scale)
2.	N-of-l trial interruption before completing three treatment pairs because of the clinician's belief that drug effectiveness had been established or refuted (perceived large treatment effect or severe side effects, both confirmed after breaking the code, or low frequency of treatment end-points)

**Table 2:** Clinical and demographic characteristics of the 21 completers/partial-completers.

Gender (male/female)	21 (13/8)
Age in years, mean (minimum/maximum)	49 (28/70)
Bronchiectasis in chest CT (unilateral/bilateral)	(15/6)
TCM syndrome differentiation (lung and spleen deficiency syndrome/Qi and Yin deficiency syndrome)	(5/16)
Concomitant medication (yes/no)	(4/17)
Baseline of the outcomes	Mean (SD)
Symptoms scores (scores)	2.78 (0.64)
CAT scores (scores)	17.33 (6.30)
24-hour sputum volume (ml)	50.05 (64.97)

**Table 3:** Comparison of the individual data (individualized decoction versus control decoction) of 15 cases who completed three pairs of the N-of-1 trials in each outcome.

Patient number	Likert scale score of symptoms mean difference and 90% CI	*P*	24-hour sputum volume mean difference and 90% CI	*P*	CAT scores mean difference and 90% CI	*P*	TCM syndrome scores mean difference and 90% CI	*P*
Case 1	−0.11 (−0.35, 0.13)	0.320	−10.00 (−26.86, 6.86)	0.225	0.17 (−4.48, 4.81)	0.926	−0.67 (−2.61, 1.28)	0.423
Case 2	0.14 (−0.23, 0.51)	0.389	−1.50 (−3.73, 0.73)	0.188	0.17 (−1.12, 1.45)	0.742	0.67 (−3.58, 4.91)	0.691
Case 3	0.06 (0.26, 0.38)	0.637	−23.33 (−65.76, 19.09)	0.250	1.33 (0.36, 2.31)	0.057	0.00 (−1.69, 1.69)	1.000
Case 4	−0.22 (−0.48, 0.05)	0.137	−4.00 (−16.16, 8.16)	0.438	−1.00 (−7.08, 5.08)	0.678	−2.33 (−7.48, 2.82)	0.317
Case 5	−0.30 (−0.88.0.29)	0.280	−1.67 (−14.54, 11.21)	0.742	1.33 (−2.07, 4.74)	0.371	−3.0 (−8.84, 2.84)	0.272
Case 6	0.35 (−0.89, 1.58)	0.497	1.667 (−8.46, 11.80)	0.678	1.33 (−4.65, 7.31)	0.582	4.00 (−5.39, 13.39)	0.339
Case 7	2.02 (−0.67, 4.71)	0.159	※	※	7.67 (0.065, 15.269)	0.099	7.67 (−0.30, 15.63)	0.107
Case 8	0.13 (−0.76, 1.01)	0.715	※	※	0.50 (−1.73, 2.73)	0.580	0.67 (−1.28, 2.61)	0.423
Case 9	0.19 (−0.40, 0.78)	0.444	7.22 (−19.78, 34.22)	0.517	1.33 (−2.66, 9.99)	0.233	5.00 (−0.84, 10.84)	0.130
Case 11	−0.08 (−1.25, 1.09)	0.859	−1.11 (−50.21, 47.99)	0.953	0.00 (−6.74, 6.74)	1.000	−0.33 (−6.25, 5.59)	0.885
Case 13	−0.11 (−0.27, 0.05)	0.184	0.56 (−7.56, 8.67)	0.860	−2.33 (−5.84, 1.18)	0.192	−1.00 (−5.46, 3.46)	0.580
Case 14	0.05 (−0.96, 1.06)	0.894	−3.33 (−29.09, 22.42)	0.742	−0.33 (−4.58, 3.91)	0.840	0.33 (−1.61, 2.28)	0.667
Case 15	0.03 (−0.34, 0.39)	0.839	−3.89 (−12.92, 5.14)	0.336	−0.67 (−1.64, 0.31)	0.184	0.33 (−2.24, 2.91)	0.742
Case 17	0.40 (−0.80, 1.61)	0.433	−6.11 (−26.44, 14.22)	0.473	0.00 (−7.35, 7.35)	1.000	3.00 (−3.08, 9.08)	0.286
Case 20	0.07 (−0.05, 0.19)	0.237	0.56 (−1.07, 2.18)	0.423	0.67 (−0.31, 1.64)	0.184	0.33 (−0.64, 1.31)	0.423

*Note.* ※ means not available.

**Table 4:** Comparison of group data between the two decoctions in each outcome.

Group level data (*n* = 21)	Individualized decoction *X* ± *S*	Individualized decoction removed of heat-clearing drugs *X* ± *S*	Mean difference and 95% CI	*t*	*P* values
Symptoms score on Likert scale	2.08 ± 0.68	1.94 ± 0.69	0.19 (0.01, 0.37)	2.20	0.040▲
Respiratory symptoms score on Likert scale	2.11 ± 0.75	1.98 ± 0.78	0.15 (−0.05, 0.35)	1.57	0.132
24-hour sputum volume (ml)	31.52 ± 40.54	33.74 ± 44.44	−1.46 (−3.59, 0.67)	−1.40	0.168
CAT scores	13.95 ± 6.97	13.66 ± 6.57	0.86 (0.042, 1.67)	2.19	0.040^*∗*^
TCM syndrome scores (lung and spleen Qi deficiency syndrome)	7.50 ± 3.12	7.75 ± 4.69	−0.73 (−5.70, 4.24)	−0.40	0.704
TCM syndrome scores (Qi and Yin deficiency syndrome)	14.51 ± 6.46	13.70 ± 6.57	1.12 (−1.23, 3.47)	1.02	0.324

*Note.* ▲ indicates that symptom scores on the Likert scale of the individualized decoction were higher than those of individualized decoction removed of heat-clearing drugs. *∗*indicates that the CAT score of individualized decoction was higher than that of the individualized decoction removed of heat-clearing drugs.

## Data Availability

All data included in this study are available upon request by contact with the corresponding author.
